# The effect of PLISSIT based counseling model on sexual function, quality of life, and sexual distress in women surviving breast cancer: a single-group pretest–posttest trial

**DOI:** 10.1186/s12905-021-01570-4

**Published:** 2021-12-16

**Authors:** Zohreh Keshavarz, Elham Karimi, Samira Golezar, Giti Ozgoli, Maliheh Nasiri

**Affiliations:** 1 Department of Midwifery and Reproductive Health, School of Nursing and Midwifery, Shahid Beheshti University of Medical Sciences, Tehran, Iran; 2 Student Research Committee, School of Nursing and Midwifery, Shahid Beheshti University of Medical Sciences, Tehran, Iran; 3 Department of Midwifery, Faculty of Nursing and Midwifery, Kermanshah University of Medical Sciences, Kermanshah, Iran; 4 Department of Basic Sciences, School of Nursing and Midwifery, Shahid Beheshti University of Medical Sciences, Tehran, Iran

**Keywords:** Counseling, PLISSIT model, Sexual dysfunction, Sexual distress, Quality of life, Breast cancer

## Abstract

**Background:**

Diagnosis and treatment of breast cancer potentially leads to sexual dysfunction and sexual distress in women and negatively affects their quality of life (QoL). This study aimed at determining the effect of PLISSIT based counseling on sexual function, sexual distress, and QoL in women surviving breast cancer.

**Methods:**

In this pre-test, post-test, single-group semi-experimental study, 65 women surviving breast cancer who were referred to the selected centers were included in the study via the available sampling method. Data gathering tools included a researcher-made demographic questionnaire, female sexual function index, beck depression inventory-II, State‐Trait Anxiety Inventory, World Health Organization QoL-Brief, and Female Sexual Distress Scale-Revised. The counseling program (7 sessions 60 min each) was designed based on the PLISSIT model. The sexual function, sexual distress, and QoL were evaluated before, and 2 and 4 weeks after the intervention. To compare the mean scores of variables before and after the intervention, repeated-measured ANOVA was used.

**Results:**

The findings showed that PLISSIT based counseling significantly reduced sexual distress and increased the scores of QoL and all its domains, as well as sexual function and all its domains in women surviving breast cancer (*p* < 0.05). There was no significant difference in the mean scores of variables 2 and 4 weeks after the intervention.

**Conclusions:**

It seems that PLISSIT based counseling reduces sexual dysfunction and sexual distress and improves the QoL of women surviving breast cancer. So, it is recommended that these counseling programs be integrated into the health care program of this group of women.

***Trial registration*:**

TCTR202103170010, 17 March 2021, Retrospectively registered, at https://www.thaiclinicaltrials.org/.

**Supplementary Information:**

The online version contains supplementary material available at 10.1186/s12905-021-01570-4.

## Background

Breast cancer is the most common cancer around the world, with an estimated 1.7 million new cancer cases diagnosed in 2012 worldwide [1, 2]. Due to an increase in life expectancy, urbanization, and following the Western lifestyle, breast cancer is rising in the developing countries [3]. With a prevalence of 18.24%, breast cancer is the most common cancer among Iranian women [4]. Because of early diagnosis and the use of effective therapeutic methods, most women with breast cancer survive [5]. In patients with breast cancer, 5-year survival rates are reported as 70–85% worldwide [6]. Annually, approximately 10,000 cases of breast cancer are diagnosed in Iran with a five-year overall survival rate of 72% [7].

Breast cancer negatively affects the QoL and patients frequently report QoL concerns about physical, psychological, social, and spiritual issues [8–10]. Because of the importance of the breast in forming the female sexual identity, the reaction against this disease can include fear, anxiety, and depression [11]. Besides, breast cancer treatments may result in some sexual problems such as sexual dysfunction, sexual distress, and unsatisfactory body image due to the loss of a body part [12, 13]. In Iran, the prevalence of these sexual dysfunctions was reported as 84% in post-treatment [14] which is close to the global records [15]. Sexual dysfunction can negatively affect the QoL and interpersonal relationships [15, 16]. Therefore, improving sexual function in these patients not only can enhance medical practice, but also can improve their well-being, QoL, and interpersonal relationships [17].

One of the most widely used interventions in the field of evaluating and managing sexual problems is the PLISSIT model. The model consists of four steps for addressing sexual concerns: Permission, Limited Information, Specific Suggestions, and Intensive Therapy [17, 18]. This model provides a framework for health care providers to implement appropriate and effective strategies to address sexual concerns [19, 20]. Health care providers have an important role in educating and providing psychological support to breast cancer patients and their families [21].

Since the survival rate of women with breast cancer is increasing due to advances in diagnostic and therapeutic methods, it is necessary to take measures to control the complications of the disease and improve their QoL [10, 21]. Considering the scarcity of research on the application of the PLISSIT model concerning sexual function, sexual distress, and QoL of breast cancer patients, the present study was conducted to determine the effect of PLISSIT- based counseling model on sexual function, QoL, and sexual distress in women surviving breast cancer.

## Methods

The present study was performed as a single group pretest–posttest semi-experimental design.

### Participant recruitment

The sample population was women surviving breast cancer referred to the gynecological clinics of selected centers in Tehran, Iran (Bu Ali and Mehr Hospital), from April to October 2017 who met the inclusion criteria. The sample size was 59 estimated following similar research [18] and using the following formula.

$$n \ge 2\frac{{\left( {z_{\alpha /2} + z_{\beta } } \right)^{2} \sigma^{2} }}{{\left( {\mu_{1} - \mu_{2} } \right)^{2} }}({1} -\uprho )$$

**Fig. 1:**
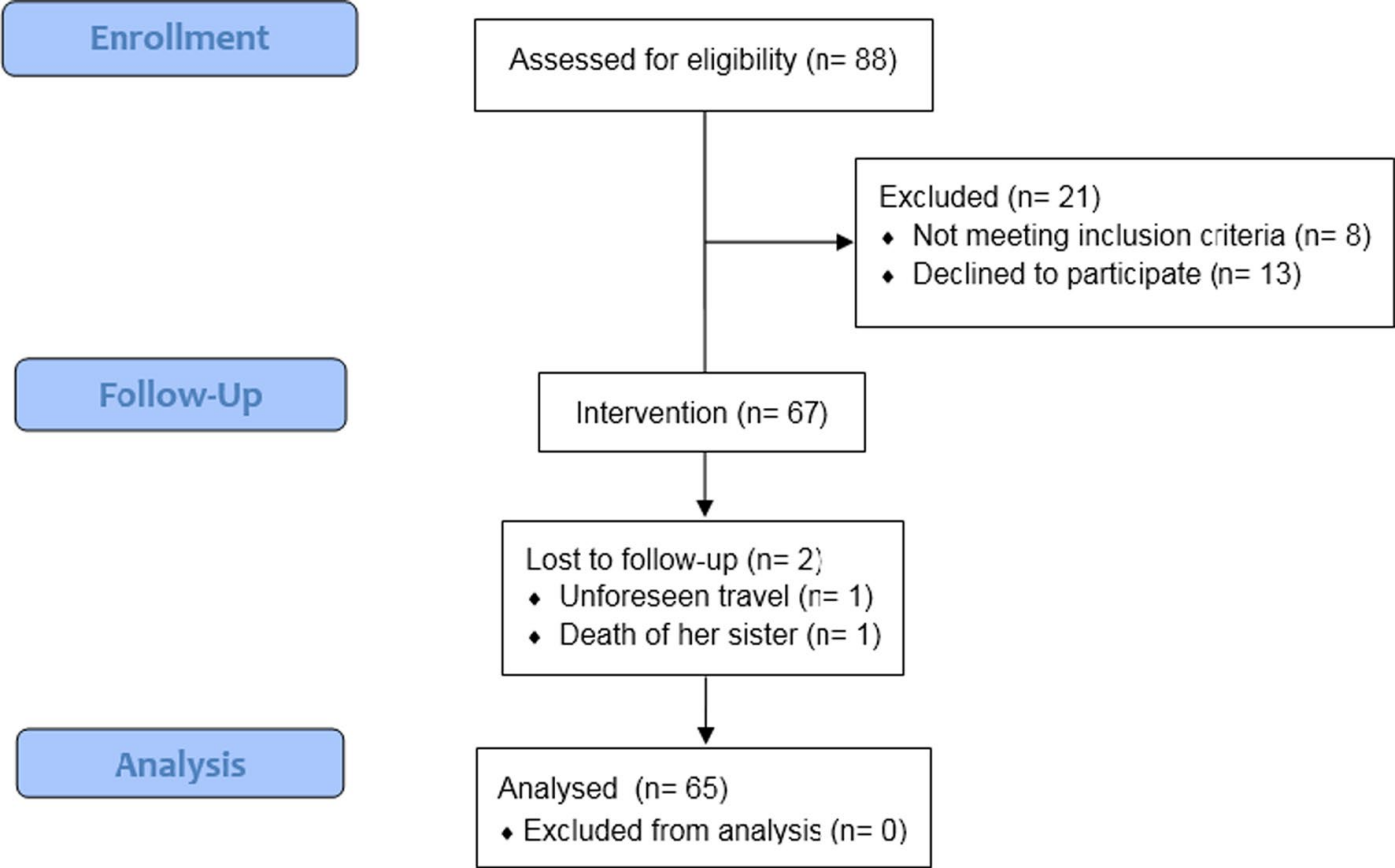
The study flowchart

Subjects were selected using the available sampling method. Finally, 67 women entered the study, and 65 of them completed the study (Fig. 1).

#### Inclusion criteria

literacy, being married, absence of chronic psychological and physical diseases, not having taken Tamoxifen for at least three years, having a history of mastectomy, having at least one sexual dysfunction (scores lower than 28 in FSFI questionnaire).

#### Exclusion criteria

Gynecological tumor such as vaginal, cervical, and uterine cancer, a history of pelvic injury that prevents sexual intercourse, alcohol or substance abuse, and the existence of sores or any lesions in the genitalia, having depression (scores higher than 13 in Beck Depression Inventory-II), and having anxiety (scores higher than 42 in State‐Trait Anxiety Inventory).

### Study tools

Data gathering tools included a researcher-made demographic questionnaire, FSFI, BDI-II, STAI, WHOQOL-BREF, and FSDS-R.

1. The researcher-made demographic questionnaire consisted of 7 questions designed according to the purpose of the study including age, duration of the marriage, woman and her husband’s education, woman and her husband’s occupation, and the number of children. the demographic questionnaire developed for this study is provided as Additional file 1.
2. The FSFI questionnaire included 19 questions that evaluate the female sexual function in 6 domains including sexual desire, arousal, lubrication, orgasm, satisfaction, and pain. The total score is the sum of the scores in all domains [22]. All items are rated on a 5-point Likert scale (1–5), The maximum score is 36, and scores lower than 28 indicate an undesirable sexual function [19]. The validity and reliability of this scale have been confirmed in several studies in Iran [23–25].
3. The BDI-II included 21 items aimed at diagnosing and measuring the severity of depression. All items are rated on a 4-point Likert scale (0–3). A score of 0–13 shows minor depression, a score of 14–19 indicates mild depression, a score of 20–28 represents moderate depression, and a score of 29–63 is a sign of severe depression [26]. The validity and reliability of this scale have also been confirmed in Iran [27].
4. The STAI is a psychological inventory that includes 40 items. Both state and trait anxiety levels are measured by this scale. They score on a 4-point Likert scale (1–4), in which higher scores show greater levels of anxiety symptoms [28]. The cut-off point on the state and trait anxiety scale is 43 and above, indicating moderate to high anxiety [29]. The validity and reliability of this scale have been confirmed in Iran [29, 30]
5. The WHOQOL-Bref questionnaire includes 26 questions that measure the QoL. This questionnaire has 4 domains i.e. physical health (7 questions), psychological (6 questions), social relationships (3 questions), and environment (with 8 questions). Also, this questionnaire has two other questions that assess general health and overall QoL. All items are rated on a 5-point Likert scale (1–5); first, a raw score is calculated for each domain which is then converted to a standard score ranging between 0 and 100. A higher score is indicative of a higher level of QoL [31]. Likewise, The reliability and validity of this questionnaire have been confirmed in Iran [32].
6. The FSDS-R questionnaire included 12 items that assess sexual distress associated with sexual activities. Each item is rated on a 5-point Likert scale (0–4). A higher score indicates greater sexual distress [33]. Like the previous questionnaires, the validity and reliability of this scale have been confirmed in Iran [34].

### Data collection

After obtaining permission from the ethics committee, the researcher (E.K) invited all the women surviving breast cancer referred to the selected centers and explained the purpose of the study and the frequency of counseling sessions to them. They were also reassured about the confidentiality of their information.

At first, the demographic, FSFI, STAI, and BDI-II questionnaires were completed by all participants and if eligible, they entered the study. After obtaining informed written consent, the consultation schedules were arranged with them. Then, the selected women answered the questionnaires of WHOQOL-BREF, FSDS-R, and FSFI in the pretest stage. In all samples, the sexual function, sexual distress, and QoL were again evaluated 2 and 4 weeks after intervention (posttest stage). The intervention included a PLISSIT-based consultation model for sexual treatment. 7 counseling sessions were conducted lasting 60 min for 4 weeks. All sessions were conducted individually in the counseling room in the selected centers. PLESSIS-based intervention is as follows:

1. Permission: researcher talked about breast cancer and sexuality during the first and second sessions. In this stage, the researcher used her counseling skills, especially listening skills and attention to nonverbal gestures, and a therapeutic rapport was established (Developing Rapport). The researcher first asked some open-ended questions about sexual function. For example, ‘what was your sexual experience when you were diagnosed with breast cancer?’. During the client's conversation, the researcher noticed her misconceptions, lack of information, and concerns, and by taking a sexual history and clinical examination, the client's sexual problem was finally identified.
2. Limited knowledge: During the third and fourth sessions, the researcher provided participants with brief information about the effects of breast cancer and its treatments on sexual function. At this stage, the researcher focused on correcting misconceptions such as ‘intercourse makes my symptoms worse’. Also, brief descriptions were given of the anatomy and physiology of the reproductive system, the sexual response cycle, and the individual's sexual problem. In this regard, 10 color images were used as auxiliary tools.
3. Specific suggestion: During the fifth and sixth sessions, the researcher used a problem-solving approach to address issues that the patient had personally experienced. For example, if a woman expressed concern about sexual intercourse with her husband for fear of pain and discomfort, the researcher recommended the use of relaxation strategies and appropriate medications (prescribed by a doctor).
4. Intensive therapy: In the seventh session, more complex cases of sexual dysfunction were referred to specialists (such as a sex therapist, psychologist, and specialist physician) for diagnosis and treatment. This stage was necessary for patients with severe or long-term sexual problems to receive an intensive and more specific treatment.

### Statistical analysis

All data were analyzed by the SPSS software version 22. The normal distribution of all continuous variables was investigated using the Kolmogorov–Smirnov test. For comparison between means of three related groups (before, and 2 and 4 weeks after intervention), repeated-measured (RM) ANOVA was used. When significant interactions were identified in the ANOVA test, the Bonferroni correction was used for post hoc comparisons. A *p*-value of 0.05 was considered significant.

## Results

65 women surviving breast cancer, with a mean age of 43.4 ± 5.6 years participated in this study. The majority of these women were 30–40 years of age. The mean duration of marriage was12.8 ± 3.9 years. The majority of the participants had a university degree and 58.49% were employed. The demographic characteristics of the participants are presented in Table 1.

**Table 1:** Demographic characteristics of the participants (n = 65)

Variable	Level	Number	Percentage
Age (years)	20–30	19	29/23
	31–40	24	36.92
	41–50	16	24.62
	51–60	6	9.23
Marriage duration (years)	5>	9	13.85
	5–10	12	18.46
	10–15	23	35.38
	15–25	21	32.31
Education level	Under diploma	5	7.7
	Diploma	20	30.77
	University degree	40	61.53
Employment status	Housewife	27	41.54
	Employee	38	58.46
Husband education	Under diploma	7	10.76
	Diploma	15	23.07
	University degree	43	66.15
Husband employment status	Employed	28	43.08
	Self-employed	26	40
	Worker	6	9.23
	Unemployed	5	7.7
Number of children	None	16	24.61
	1–2	23	35.38
	3–4	21	32.30
	5 ≤	5	7.7

According to the results, the mean scores of total sexual function and QoL increased after the intervention. Also, the mean score of sexual distress decreased after the intervention and all these changes were statistically significant (*p* < 0.001). Table 2 shows the changes in sexual distress, total sexual function, and QoL at the three measurement points. The findings of the Bonferroni post hoc test showed a statistically significant difference for each of these variables before and after the intervention (*p* < 0.001). However, there was no significant difference between the mean scores of variables 2 weeks and 4 weeks after the intervention (*p* > 0.05).

**Table 2:** Changes of sexual distress, total sexual function, and QoL over time among women who received PLISSIT-based intervention (n = 65)

Variable	Before intervention (T1)	2 weeks after intervention (T2)	4 weeks after intervention (T3)	F	*p*-value*	*p*-value** (pairwise comparison)
	Mean (SD)	Mean (SD)	Mean (SD)			
Sexual function	23.33 (2.72)	31.66 (3.77)	31.44 (3.88)	11.8	0.001	T1 < T2 (*p* = 0.001) T1 < T3 (*p* = 0.001)
Sexual distress	24.34 (4.21)	21.74 (4.16)	21.52 (4.08)	9.92	0.001	T1 > T2 (*p* = 0.003) T1 > T3 (*p* = 0.001)
QoL	51.63(4.14)	58.35 (8.23)	58.91 (7.25)	15.6	0.001	T1 < T2 (*p* = 0.001) T1 < T3 (*p* = 0.001)

^*^Repeated measure ANOVA test

^**^Bonferroni posthoc test

Also, the results of RM-ANOVA showed that the scores of different dimensions of sexual function and QoL after counseling increased significantly (*p* < 0.001), which are shown in Tables 3 and 4, respectively. Pairwise comparison by Bonferroni post hoc test showed significant differences between the mean scores of variables before and after the intervention for all domains of QoL and sexual function (*p* < 0.001), but there was no significant difference between the mean scores of variables 2 and 4 weeks after the intervention (*p* > 0.05).

**Table 3:** Changes of main domains of sexual function over time among women who received PLISSIT-based intervention (n = 65)

Variable	Before intervention (T1)	2 weeks after intervention (T2)	4 weeks after intervention (T3)	F	*p*-value*	*p*-value** (pairwise comparison)
	Mean (SD)	Mean (SD)	Mean (SD)			
Desire	3.12 (0.92)	3.97 (1.01)	4.15 (1.43)	15.25	0.001	T1 < T2 (*p* = 0.001) T1 < T3 (*p* = 0.001)
Arousal	5.57 (1.95)	7.54 (2.29)	6.86 (2.35)	13.13	0.001	T1 < T2 (*p* = 0.001) T1 < T3 (*p* = 0.001)
Lubrication	4.41 (0.93)	5.66 (1.62)	5.69 (2.33)	10.99	0.001	T1 < T2 (*p* = 0.001) T1 < T3 (*p* = 0.001)
Orgasm	3.67 (1.10)	5.06 (1.57)	5.29 (1.86)	18.93	0.001	T1 < T2 (*p* = 0.001) T1 < T3 (*p* = 0.001)
Satisfaction	3.36 (1.45)	4.91 (1.43)	5.09 (1.53)	28.10	0.001	T1 < T2 (*p* = 0.001) T1 < T3 (*p* = 0.001)
Pain	3.18 (1.23)	4.52 (1.65)	4.35 (1.96)	12.15	0.001	T1 < T2 (*p* = 0.001) T1 < T3 (*p* = 0.001)

^*^Repeated Measure ANOVA test

^**^Bonferroni post-hoc test

**Table 4:** Changes of main domains of QoL over time among women who received PLISSIT-based intervention (n = 65)

Variable	Before intervention (T1)	2 weeks after intervention (T2)	4 weeks after intervention (T3)	F	*p*-value	*p*-value** (pairwise comparison)
	Mean (SD)	Mean (SD)	Mean (SD)			
Physical health	13.88 (1.71)	15.15 (1.84)	15.31 (1.88)	18.37	0.001	T1 < T2 (*p* = 0.001) T1 < T3 (*p* = 0.001)
Psychological	12.08 (2.14)	13.75 (3.98)	13.91 (2.65)	11.36	0.001	T1 < T2 (*p* = 0.001) T1 < T3 (*p* = 0.001)
Relationships	6.29 (1.07)	7.20 (1.91)	7.31 (1.95)	10.10	0.001	T1 < T2 (*p* = 0.001) T1 < T3 (*p* = 0.001)
Environment	15.74 (2.04)	17.94 (2.69)	17.69 (2.96)	21.37	0.001	T1 < T2 (*p* = 0.001) T1 < T3 (*p* = 0.001)

^*^Repeated Measure ANOVA test

^**^Bonferroni posthoc test

## Discussion

The present study was a quasi-experimental pre-test and post-test one-group study that aimed to determine the effect of the PLISSIT-based consultation model on sexual function and sexual distress and QoL of 65 women surviving breast cancer.

In the current study the mean age of participants was 43.4 ± 5.6 years which matched the mean age of participants of Saboula and Shahin [18], and Faghani and Ghaffari [35] studies, i.e., 43.1 ± 10.0 years and 43.2 ± 4.6 years respectively. Also, 60% of women in the present study were below 40 years of age that is similar to other studies [15, 18, 36]. The age distribution of breast cancer in Iranian women is about one decade lower than other countries, which indicates more psychological and sexual problems compared to others [36].

The results showed that PLISSIT-based counseling significantly reduced sexual distress and increased the scores of QoL and all its domains, and sexual function and all its domains in women with breast cancer. However, there was no significant difference in the mean scores of variables between 2 and 4 weeks after the intervention, which indicates the reliability of the intervention.

Consistent with our study, Faghani and Ghaffari showed improved quality of sexual life and sexual function and its all aspects in post-mastectomy women four weeks after the PLISSIT- based consultation presented in four 90-min sessions [35]. Esmkhani et al., reported similar effects on improving all domains of QoL of women with breast cancer 6, and 12 weeks after application of PLISSIT model [36]. Also, Saboula and Shahin showed that the application of the PLISSIT model was effective in enhancing body image, couple satisfaction, and all sexual function index domains except for the domain of desire for women with breast cancer three weeks after receiving six 2 h counseling sessions [18]. The influence of various factors such as fatigue, stress, depression, and couple relationships on sexual desire can justify this difference with our study [37]. Other studies have found similar results on the effect of PLISSIT-based counseling on improving sexual function, sexual distress, and marital intimacy in women with gynecological cancers [38, 39]. Contrary to these results, in a study by Khoei et al., consultation using the PLISSIT model had no significant effect on sexual behavior of women with breast cancer compared to the control group at six weeks and 12 weeks after intervention [15]. This difference may be due to varying approaches to implementing interventions, the number of sessions, tools, and characteristics of the study population.

One of the limitations of our study was the absence of the participants' husbands. Second, due to the limited access to samples, it was not possible to conduct a clinical trial with the control group. Third, the participants were selected from large urban areas, which might hamper generalizability of the results.

## Conclusion

In general, the increasing prevalence of breast cancer in recent years and its negative effects on the physical, psychological and social aspects of women's lives has led to its being recognized as a major women health problem that has a negative effect on their QoL through reducing sexual function and distress [15, 16]. Besides, there is a lack of adequate related education, so, educating women surviving breast cancer on changes in sexual function following cancer through intervention programs such as PLISSIT based counseling can help them cope with the disease and thus improve these women's QoL as an important member of the family. Therefore, due to the simplicity and applicability of the PLISSIT model, it is recommended that it be integrated into the health care program (e.g. sexual health counseling and training programs) of this group of patients by health care providers such as midwives.

## Supplementary Information

**Additional file 1.** The researcher-made demographic questionnaire. This questionnaire consists of 7 items about the demographic characteristics of the participants.

## Data Availability

The datasets used and/or analysed during the current study are available from the corresponding author on reasonable request. All supporting data are available through the corresponding author.

## Abbreviations

PLISSIT: Permission, Limited Information, Specific Suggestion and Intensive Therapy
QoL: Quality of life
RM-ANOVA: Repeated measured analysis of variance
FSFI: Female sexual function index
BDI-II: Beck depression inventory-II
STAI: State‐Trait Anxiety Inventory
WHOQOL-BREF: World Health Organization Quality of Life Questionnaire-Brief
FSDS-R: Female Sexual Distress Scale-Revised
