# Supplementary material for: The effect of PLISSIT based counseling model on sexual function, quality of life, and sexual distress in women surviving breast cancer: a single-group pretest–posttest trial
Source: BMC Womens Health. 2021 Dec 16;21:417. doi: 10.1186/s12905-021-01570-4 (PMC8680023; doi:10.1186/s12905-021-01570-4)
Supplement: Supplementary file 1 — Additional file 1. The researcher-made demographic questionnaire. This questionnaire consists of 7 items about the demographic characteristics of the participants. [file 12905_2021_1570_MOESM1_ESM.docx]

**The researcher-made demographic questionnaire**

1. How old are you?

2. How many years have you been married?

Less than 5 years 5-10 years 10-15 years 15-25 years

3. What is your level of Education?

Under diploma Diploma University degree

4. What is your employment status?

Housewife Employee

5. What is your husband's level of education?

Under diploma Diploma University degree

6. What is your husband's employment status?

Employed Self-employed Worker Unemployed

7. How many children do you have?

I do not have 1 or 2 3 or 4 5 or more than
